# Dysregulation of Th17 Cells during the Early Post-Transplant Period in Patients under Calcineurin Inhibitor Based Immunosuppression

**DOI:** 10.1371/journal.pone.0042011

**Published:** 2012-07-25

**Authors:** Byung Ha Chung, Kyoung Woon Kim, Bo-Mi Kim, Shang Guo Piao, Sun Woo Lim, Bum Soon Choi, Cheol Whee Park, Yong-Soo Kim, Mi-La Cho, Chul Woo Yang

**Affiliations:** 1 Convergent Research Consortium for Immunologic Disease, Seoul St. Mary's Hospital, College of Medicine, The Catholic University of Korea Seoul, Seoul, Republic of Korea; 2 Transplant Research Center, Seoul St. Mary's Hospital, College of Medicine, The Catholic University of Korea Seoul, Seoul, Republic of Korea; 3 Division of Nephrology, Department of Internal Medicine, Seoul St. Mary's Hospital, College of Medicine, The Catholic University of Korea Seoul, Seoul, Republic of Korea; French National Centre for Scientific Research, France

## Abstract

Accumulating evidence suggests that Th17 cells play a role in the development of chronic allograft injury in transplantation of various organs. However, the influence of current immunosuppressants on Th17-associated immune responses has not been fully investigated. We prospectively investigated the changes in Th17 cells in peripheral blood mononuclear cells (PBMCs) collected before and 1 and 3 months after KT in 26 patients and we investigated the suppressive effect of tacrolimus on Th17 in vitro. In the early posttransplant period, the percentage of Th17 cells and the proportion of IL-17-producing cells in the effector memory T cells (TEM) were significantly increased at 3 months after transplantation compared with before transplantation (P<0.05), whereas Th1/Th2 cells and TEM cells were significantly decreased. The degree of increase in Th17 during the early posttransplant period was significantly associated with allograft function at 1 year after transplantation (r = 0.4, P<0.05). In vitro, tacrolimus suppressed Th1 and Th2 cells in a concentration-dependent manner, but did not suppress Th17 cells even at high concentration. This suggests that current immunosuppression based on tacrolimus is inadequate to suppress Th17 cells in KTRs, and dysregulation of Th17 may be associated with the progression of CAD.

## Introduction

After kidney transplantation, alloimmune responses by CD4+ T cell activation mediate most cases of allograft rejection [1]. Therefore, most immune suppressants have been developed to downregulate the activation and differentiation of effector CD4+ T cells to prevent the generation of these alloimmune responses [2], [3]. The most widely used immunosuppressive protocol is composed of tacrolimus (Tac), mycophenolate mofetil (MMF) and steroids. In addition, induction therapy with basiliximab has been used to suppress the robust T cell activation developed immediately after kidney transplantation.

The Tac-based immunosuppressive protocol has been shown to be more effective in preventing the development of acute rejection episodes and improving 1 year allograft survival than the previously used regimen based on azathioprine and steroids [4]. During the early posttransplant period, in which most robust immune reactions develop, most effector T cell subsets are effectively suppressed by the current immunosuppressive protocol, which may result in the significant improvement in short-term clinical outcomes. In contrast, long-term allograft survival has not improved significantly compared with that in the azathioprine era [5], [6].

Th17 is a third subset of effector T cells and is characterized by the secretion of the proinflammatory cytokine IL-17 [7], [8]. Ongoing studies have demonstrated that Th17 cells are involved in the driving of immune processes previously thought to be exclusively Th1 mediated [9], [10], [11], [12]. In addition, accumulating evidence suggests that Th17 cells may play a role in the development of chronic allograft injury in various types of organ transplantation [13], [14], [15], [16], [17]. However, the effect of Tac on Th17-associated immune responses has not been fully investigated.

In this study, we prospectively and sequentially investigated the changes in immune cell subsets, including Th17, in renal transplant recipients during the early posttransplant period, during which most significant changes in immune reactivity develop, and evaluated the relationship of the change in Th17 cells with 1-year allograft function. Second, we investigated the percentage of Th17 cells in another renal transplant recipient group with long-term follow-up, comparing patients with normal allograft function and those with chronic allograft dysfunction (CAD). Third, we tested the effect of Tac on each effector T cell subset in vitro.

## Materials and Methods

### Patients and clinical information

**Figure 1:**
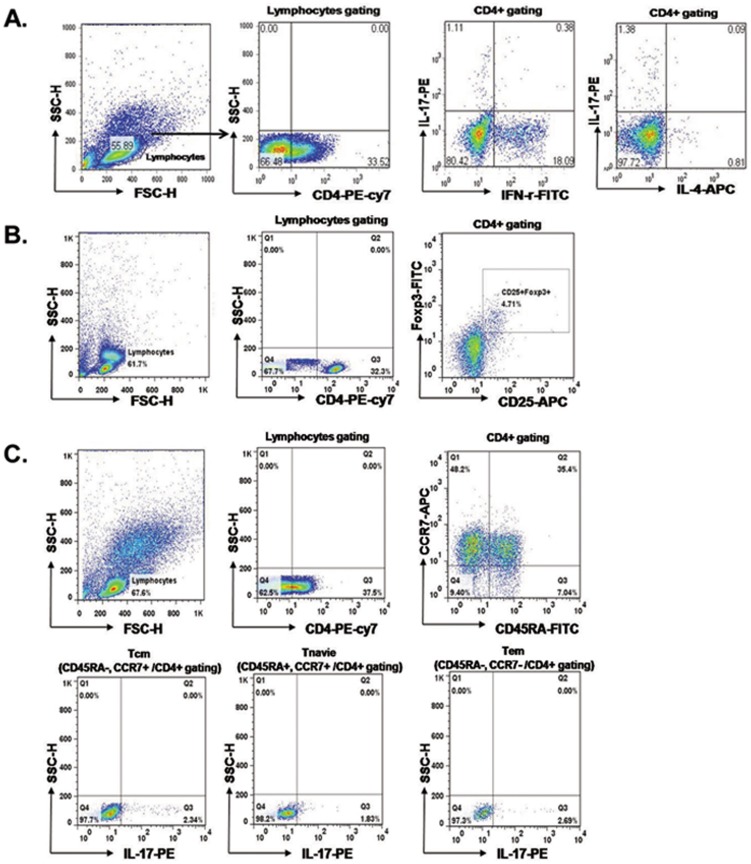
Flow cytometric analysis of T cell subsets. PBMCs were stained with anti-CD4 PE-cy7, anti-CD25 APC, anti-IFN-γ FITC, anti-IL-17 PE, anti-IL-4 APC and anti-Foxp3 FITC. CD4+ cells were gated for further analysis. PBMC from patients before KT, patients at 1month after KT and patients at 3 month after KT were stimulated for 4 h ex vivo with PMA and ionomycin in the presence of Golgi Stop. The percentage of Target cells was measured by flowcytometry. The frequency (%) of Lymphocyte/Leukocyte cells, CD4^+^T/Lymphocyte cells, IL-17+/CD4^+^T cells, IFN-γ^+^/CD4^+^T cells, IL-4^+^/CD4^+^T cells (A) and CD25^+^FOXP3^+^/CD4^+^T cells (B) in patients before KT, patients at 1month after KT and patients at 3 month after KT. After surface staining with anti-CD4, CD45 and CCR7 mAbs, cells were fixated and permeabilized and intracellular accumulated cytokines were detected with IL-17 mAbs. T_naïve_/CD4^+^T (CD45RA*^+^*CCR7^+^/CD4^+^Tcells), IL-17^+^/T_naïve_, T_CM_/CD4^+^ T (CD45RA^−^CCR7^+^/CD4^+^Tcells), IL-17^+^/T_CM_^+^ and T_EM_/CD4^+^T (CD45RA^−^CCR7^−^/CD4^+^Tcells), IL-17^+^/T_EM_^+^ (C).

The patient population comprised of 26 living-donor renal transplant recipients (RTRs). Second transplants, highly sensitized patients and ABO-incompatible kidney transplants were excluded from this study because they were treated with modified or intensified immune suppressant protocols. For the included patients, the initial immunosuppressant was Tac in combination with MMF and prednisolone. Basiliximab was used as additional induction therapy at 2 hours before transplantation and on day 4 after transplantation. The initial dose of Tac was 0.16 mg/kg per day orally, and target trough levels were 8–12 ng/mL during the first 3 months and 3–8 ng/mL afterward (Figure 1). Methylprednisolone (1 g/day) was administered by intravenous infusion on the day of transplantation and was tapered to prednisolone at 30 mg/day on day 4 after transplantation. The initial dose of MMF was 1.5 g/day and the dose was modified to minimize adverse effects such as diarrhea or leukopenia. Peripheral blood mononuclear cells (PBMC) were collected for the analysis before the initiation of immunosuppressive treatment, and at around 1 month and 3 months after transplantation.

We followed all RTRs for at least 1 year from transplantation. We calculated the differences between the Th17 percentage before transplantation and at 3 months post transplant, calling this ΔTh17. We evaluated whether ΔTh17 had a significant association with the 1-year allograft function to investigate the clinical impact of the changes in Th17 during the early post-transplant period.

We enrolled another patient group who were being treated with Tac (n = 16) with a long-term follow-up (9.5 years (5.1–13.1)) and who showed stable allograft function without significant change (>20% from baseline) and no acute rejection for at least the past 1 year. These patients comprised two groups that were matched for age and post-transplant duration. One group, designated as the no CAD group (n = 8), was comprised of 8 patients who have showed favorable allograft function (MDRD eGFR higher than 60 mL/min/1.73 m^2^) since KT and have showed the change of eGFR within 20% of the value at 1 year from KT. Another 8 patients who have showed gradually deteriorated allograft function since KT (decrease of eGFR more than 20% compared to the value at 1 year from KT) and MDRD eGFR at enrollment was lower than 60 mL/min/1.73 m^2^. All patients in CAD group took at least 1 time of allograft biopsy during follow up and allograft tissue showed chronic change (Interstitial fibrosis/Tubular atrophy score (IF/TA) ≥2). We compared the proportion of Th1, Th2 and Th17 cells in the CD4^+^ T cell populations of the two groups to investigate the clinical impact of Th17 on chronic allograft injury. This study was approved by the Institutional Review Board (KC10SISI0235) of the Seoul St. Mary's Hospital, and we obtained written informed consent from all patients and all clinical investigations have been conducted according to the principles expressed in the Declaration of Helsinki.

### Isolation of human cells

PBMC were prepared from heparinized blood by Ficoll–Hypaque (GE Healthcare, PA) density-gradient centrifugation. Cell cultures were performed as described previously [18]. In brief, the cell suspension was adjusted to a concentration of 10^6^/ml in RPMI1640 medium supplemented with 10% fetal calf serum, 100 U/mL penicillin, 100 mg/mL streptomycin, and 2 mM l-glutamine. The cell suspension (1 mL) was dispensed into 24-well multiwell plates (Nunc, Roskilde, Denmark). For cytokine detection at the single-cell level, PBMC were stimulated with 50 ng/mL phorbol myristate acetate (PMA) and 1 µg/mL ionomycin for 4 h.

### Flow cytometric analysis

Flowcytometic study of PBMC was done within a few hours after the sampling of peripheral blood as a fresh state. For analysis of human intracellular cytokine production, PBMC were stimulated with PMA and ionomycin in the presence of GolgiStop (BD Biosciences, San Diego, CA) for 4 hours. For intracellular staining, cells were stained with combinations of the following mAbs: CD4–PE/Cy7 (RPA-T4, IgG1; BioLegend, San Diego, CA); CD45RA–FITC (HI100, IgG2b, κ; Pharmingen, San Diego, CA) and CD25–APC (M-A251, IgG1, κ; Pharmingen). Staining for chemokine receptors was performed using the following mouse mAbs (all produced by Pharmingen): anti-CCR4 (1G1, IgG1), anti-CCR6 (11A9, IgG1) and anti-CCR7 (3D12, IgG2a, κ). Cells were washed, fixed, permeabilized and stained to detect intracellular cytokines with mAbs to IL-17 (PE, eBio64dec17, IgG1, κ; eBioscience, San Diego, CA); interferon (IFN)-γ (FITC, 4S.B3, IgG1, κ; eBioscience); IL-4 (APC, MP4-25D2, IgG1, κ; eBioscience); IL-17 (FITC (eBio64DEC17, IgG1, κ; eBioscience); Foxp3 (FITC, PCH101, IgG2a, κ; eBioscience) and IFN-γ (PE, B27, IgG1, κ; Pharmingen). Appropriate isotype controls were used for gate setting for cytokine expression. Cells were analyzed on a FACS Calibur flow cytometry system (BD Biosciences).

### Real-time reverse transcription polymerase chain reaction (RT-PCR)

After incubation of PBMC for 4 h with PMA and ionomycin, mRNA was extracted using RNAzol B (Biotex Laboratories, Houston, TX) according to the manufacturer's instructions. Reverse transcription of 2 µg of total mRNA was performed at 42°C using the Superscript™ reverse transcription system (Takara, Shiga, Japan). Polymerase chain reaction (PCR) was performed in a 20 µL final volume in capillary tubes in a LightCycler instrument (Roche Diagnostics, Mannheim, Germany). The reaction mixture contained 2 µL of LightCycler Fast Start DNA Master Mix for SYBR® Green I (Roche Diagnostics), 0.5 µM of each primer, 4 mM MgCl_2_ and 2 µL of template DNA. All capillaries were sealed, centrifuged at 500×*g* for 5 s, and then amplified in a LightCycler instrument with activation of polymerase (95°C for 10 min), followed by 45 cycles of 10 s at 95°C, 10 s at 60°C (β-actin) or 57°C (IL-1beta, HMGB-1), and 10 s at 72°C. The temperature transition rate was 20°C/s for all steps. The double-stranded PCR product was measured during the 72°C extension step by detection of fluorescence associated with the binding of SYBR Green I to the product. Fluorescence curves were analyzed with LightCycler software v. 3.0 (Roche Diagnostics). The LightCycler was used to quantify IL-1beta and HMGB-1 mRNA. The relative expression level of each sample was normalized to the endogenously expressed housekeeping gene (β-actin). Melting curve analysis was performed immediately after the amplification protocol under the following conditions: 0 s (hold time) at 95°C, 15 s at 71°C, and 0 s (hold time) at 95°C. The rate of temperature change was 20°C/s, except for 0.1°C/s in the final step. The crossing point (*C*_p_) was defined as the maximum of the second derivative from the fluorescence curve. Negative controls were also included and contained all the elements of the reaction mixture except template DNA. All samples were processed in duplicate.

### Suppressive effect of TAC on Th0 or Th17 cells in vitro

Isolated PBMC cells (5×10^5^) from healthy people were incubated under appropriate conditions to stimulate Th0 cells for 48 h. Anti-CD3 (1 µg/mL) and anti-CD28 (1 µg/mL) were used to differentiate Th0 cells. Anti-CD3 (1 µg/ml), anti-CD28 (1 µg/ml), IL-1b (20 ng/ml), IL-6 (20 ng/ml) and IL-23 (20 ng/ml) were added to stimulate the differentiation of Th17 cells. For analysis of the suppressive effect of Tac, PBMC from healthy people and renal transplant recipients were preincubated for 1 h in the presence of Tacrolimus and stimulated with Th0 or Th17-polarizing condition for 48 h. Neutralizing antibodies to IFN-gamma (2 µg/ml) and IL-4 (2 µg/ml) were added in some experiments (R&D Systems).

### Statistical analysis

Statistical analysis was performed using SPSS software (version 16.0; SPSS Inc., Chicago, IL). The comparison of values before transplantation and at 1 month and 3 months after transplantation was made using a paired *t*-test or one-way analysis of variance. For categorical variables, chi-square frequency analysis was used. The results are presented as mean ± standard deviation (SD). *P* values<0.05 were considered significant.

## Results

### Baseline characteristics of patient population

The age at transplantation was 42.8±11.6 years. Eighteen patients were male and eight female. The HLA mismatch number was 3.2±1.8 and all cases were first kidney transplants. Thirteen patients (50.0%) were on hemodialysis and five patients (19.2%) on peritoneal dialysis before transplantation and the duration of dialysis was 13.7±18.8 months. Eight patients (30.8%) took preemptive transplantation. The most common cause of end-stage renal disease was chronic glomerulonephritis (11 patients, 42.3%) followed by diabetes mellitus (five patients, 19.2%), hypertension (five patients, 19.2%). Eighteen cases (69.2%) were living-related donor transplants and eight (30.8%) were living-unrelated donor transplants (Table 1).

**Table 1:** Baseline characteristics of patient population in prospective observation group.

	N = 26
**Age (year)**	42.8±11.6
**Male (n, %)**	18 (69.2)
**HLA mismatch number**	3.2±1.8
**Dialysis modality before KT (n, %)**	
Hemodialysis	13 (50)
Peritoneal dialysis	5 (19.2)
Preemptive KT	8 (30.8)
**Dialysis duration (month)**	13.7±18.8
Primary renal disease (n,%)	
Chronic GN	11 (42.4)
Diabetes mellitus	5 (19.2)
Hypertension	5 (19.2)
Others	5 (19.2)
**Donor type (n, %)**	
Living related donor	18 (69.2)
Living unrelated donor	8 (30.8)

KT, kidney transplantation, GN, glomerulonephritis.

### Changes in lymphocytes and total CD4+ T cells at 1 month and 3 months after transplantation

**Figure 2:**
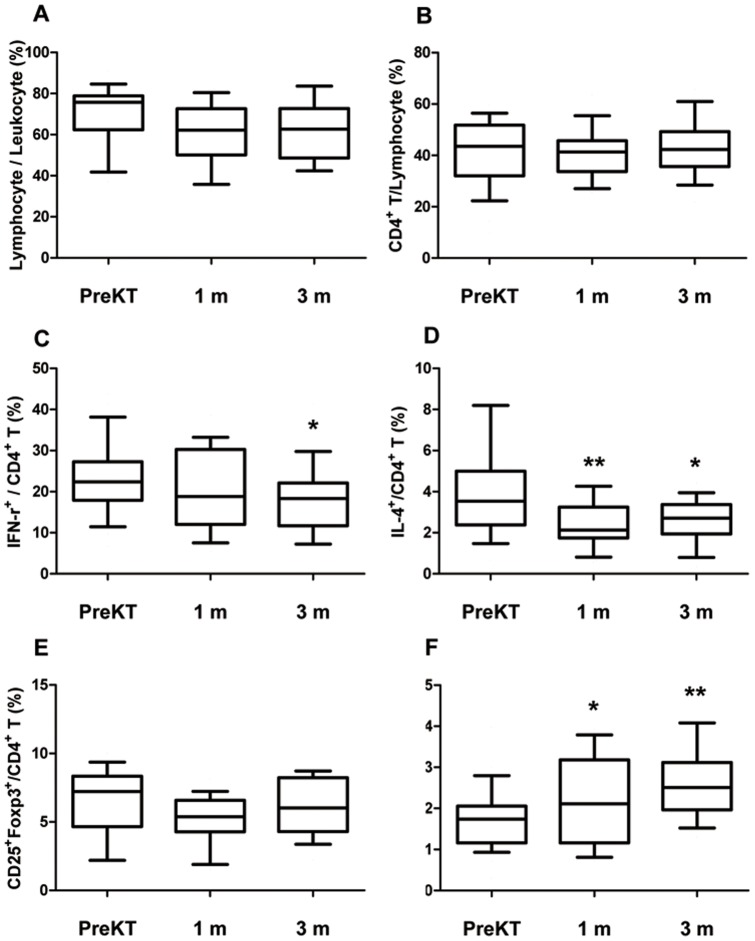
Distribution of lymphocyte and CD4+ T cell subtype at 1 and 3 month after transplantation compared to before transplantation. PBMC from patients before KT, patients at 1month after KT and patients at 3 month after KT were stimulated for 4 h ex vivo with PMA and ionomycin in the presence of Golgi Stop. The percentage of Target cells was measured by flowcytometry. The frequency (%) of Lymphocyte/Leukocyte cells (A), CD4^+^T/Lymphocyte cells (B), IFN-γ^+^/CD4^+^T cells (C), IL-4^+^/CD4^+^T cells (D) CD25^+^FOXP3^+^/CD4^+^T cells (E) and IL-17^+^/CD4^+^ T cells (F) in patients before KT, patients at 1month after KT and patients at 3 month after KT. Bars show the means. * *P*<0.05 vs. PreKT, ** *P*<0.01 vs. PreKT PreKT, before kidney transplantation; m, month.

As shown in Figure 1, Figure 2, there was a decrease in the percentage of lymphocytes after transplantation (1 month: 60.0±15.3%; 3 months: 64.1±13.6%) compared with before transplantation (70.5±15.0%), but this did not reach statistical significance (*P*>0.05) (Figure 2A). The percentage of CD4^+^ T cells showed no significant change at 1 month (41.2%±10.0%) and 3 months (43.7±11.6%) after transplantation compared with before transplantation (39.8±13.2%, *P*>0.05) (Figure 2B).

### Changes in Th1, Th2 and regulatory T cell subsets at 1 month and 3 months after transplantation

Before transplantation, 23.3±8.8% of CD4^+^ T cells were also IFN-γ^+^ (Th1). At 1 month after transplantation, this had not changed significantly (19.8±9.6%, *P* = 0.111), but after 3 months, it had significantly decreased to 17.9±9.1% (*P* = 0.02 vs. before transplant) (Figure 2C). The percentage of IL-4^+^/CD4^+^T cells (Th2) was 3.6±1.8% before transplantation, and decreased significantly to 2.4±1.2% at 1 month after transplantation (*P* = 0.007) and 2.6±1.0% at 3 months after transplantation (P = 0.04) (Figure 2D). The percentage of CD25^+^FoxP3^+^/CD4^+^ T cells (Treg) did not change at either 1 month (5.2±1.7%, *P* = 0.07) or 3 months (5.9±2.1%, *P* = 0.484) after transplantation compared with before transplantation (6.5±2.6%) (Figure 2E). The percentage of IL-17^+^/CD4^+^ T cells (Th17) significantly increased after transplantation. Before transplantation, it was 1.9±0.7% and it increased to 2.2±1.0% (*P* = 0.04) at 1 month and 2.6±0.8% at 3 months (*P* = 0.008) after transplantation (Figure 2F).

### Changes in Th17 and IL-17-related T cell subsets at 1 month and 3 months after transplantation

**Figure 3:**
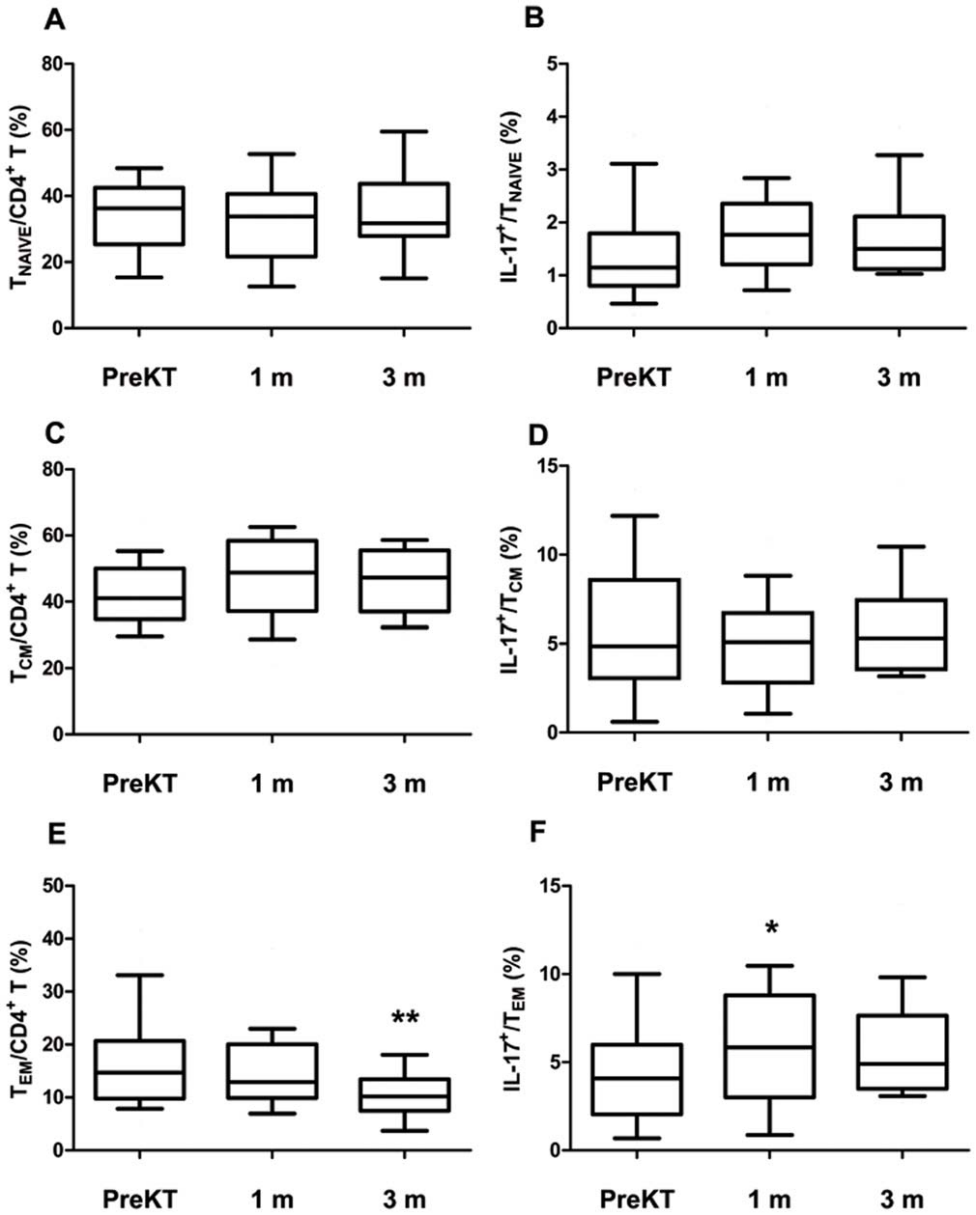
Distribution of T_naïve_, T_CM_, T_EM_ subpopulations and IL-17^+^/T_naïve_, IL-17^+^/T_EM_ and IL-17^+^/T_CM_, subpopulations of CD4^+^T lymphocytes at 1 and 3 month after transplantation compared to before transplantation. PBMC from patients before KT, patients at 1month after KT and patients at 3 month after KT were stimulated for 4 h ex vivo with PMA and ionomycin in the presence of GolgiStop.CD4^+^ lymphocytes were stained with mAbs to CD45RA and CCR7, which identified three subsets. In addition, analysis of IL-17 in CD4^+^ T cell subsets by intracellular flow cytometry was done. After surface staining with CD45 and CCR7 mAbs, cells were fixated and permeabilized and intracellular accumulated cytokines were detected with IL-17 mAbs. (A) T_naïve_/CD4^+^T (CD45RA*^+^*CCR7^+^/CD4^+^Tcells), (B) IL-17^+^/T_naïve_, (C) T_CM_/CD4^+^ T (CD45RA^−^CCR7^+^/CD4^+^Tcells), (D) IL-17^+^/T_CM_^+^, (E) T_EM_/CD4^+^T (CD45RA^−^CCR7^−^/CD4^+^Tcells), (F) IL-17^+^/T_EM_^+^. Bars show the means. * *P*<0.05 vs. PreKT, ** *P*<0.01 vs. PreKT PreKT, before kidney transplantation; m, month.

Before transplantation, the percentage of CCR7^+^CD45RA^+^/CD4^+^ T cells (T_naive_/CD4^+^ T cells) was 34.1±10.3% and this did not change significantly at 1 month (32.3±11.1%, *P* = 0.702) and 3 months (35.3±12.3%, *P* = 0.594) after transplantation (Figure 3A). CCR7^+^CD45RA^−^/CD4^+^ T cell (T_CM_/CD4^+^ T cells) also did not change significantly after transplantation (T_CM_: 42.4±9.8% before transplantation, 46.7±12.2% at 1 month after transplantation, *P* = 0.298, 46.9±9.9% at 3 months, *P* = 0.257) (Figure 3C). In contrast, CCR7^−^CD45RA^−^/CD4^+^ T cells (T_EM_/CD4^+^ T cells) were significantly decreased after transplantation (17.0±9.3% before transplantation, 14.8±7.5% at 1 month after transplantation, *P* = 0.064, 10.9±5.0% at 3 months, *P* = 0.002) (Figure 3E). The percentage of IL-17-producing cells in T_CM_ and T_naive_ showed no significant change at 1 month (T_CM_: 7.0±5.7%, *P* = 0.32; T_naive_: 1.8±0.9%, *P* = 0.15) and 3 months (T_CM_: 5.7±3.7%, *P* = 0.84; T_naive_: 1.74±0.8%, *P* = 0.20) after transplantation compared with before transplantation (T_CM_: 5.7±4.2%, T_naive_: 1.4±0.9%) (Figure 3B, 3D). The percentage of IL-17 producing cells in T_EM_ was 4.2±3.2% before transplantation and increased to 6.7±3.2% at 1 month (*P* = 0.021) and 3 months (6.1±3.7%, *P* = 0.353) after transplantation (Figure 3F).

### Change in expression of markers associated with the development of Th17 cells at 1 month and 3 months after transplantation

**Figure 4:**
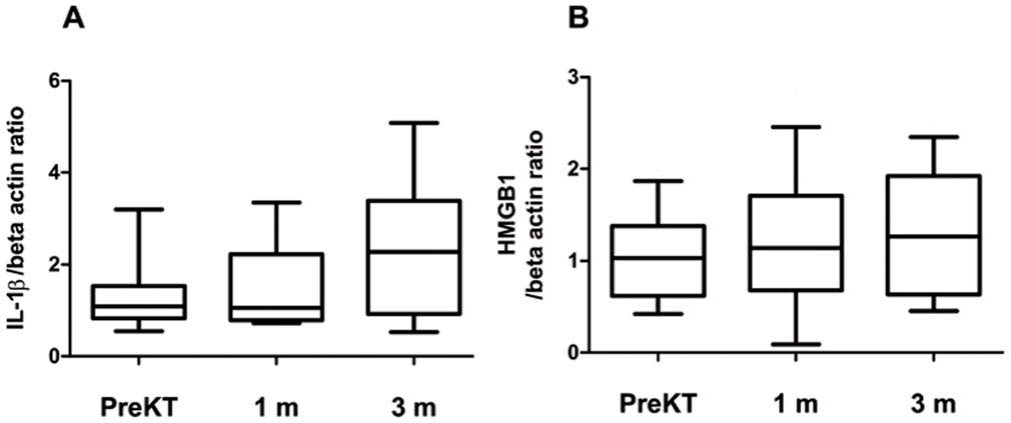
Expression of IL-1beta, and HMGB1 associated with Th17 cell at 1 and 3 month after transplantation compared to before transplantation. PBMC from patients before KT, patients at 1month after KT and patients at 3 month after KT were stimulated for 4 h ex vivo with PMA and ionomycin in the presence of GolgiStop. PBMC from all groups were treated as described in Figure 1 and Materials and Methods. The expression of IL-1beta (A), HMGB1(B) mRNA was measured using real-time PCR. Bars show the means.

After PBMC were stimulated with PMA and ionomycin, the expression of mRNA for IL-1beta and HMGB1 was determined using real-time polymerase chain reaction. As shown in Figure 4, the expression of IL-1beta mRNA was not suppressed at 1 month (1.5±0.9) and 3 months (2.4±1.6) after transplantation compared with before transplantation (1.3±0.8) but rather showed a tendency to increase (at 1 month, *P* = 0.612; at 3 months, *P* = 0.09) (Figure 4A). The expression of HMGB1 mRNA also showed a trend to increase at 1 month (1.2±0.8) and 3 months (1.3±0.7) after transplantation compared with before transplant (1.1±0.5) (at 1 month *P* = 0.60; at 3 months, *P* = 0.36) (Figure 4B).

### Relationship of long-term allograft function with the changes in immune cell subsets

**Figure 5:**
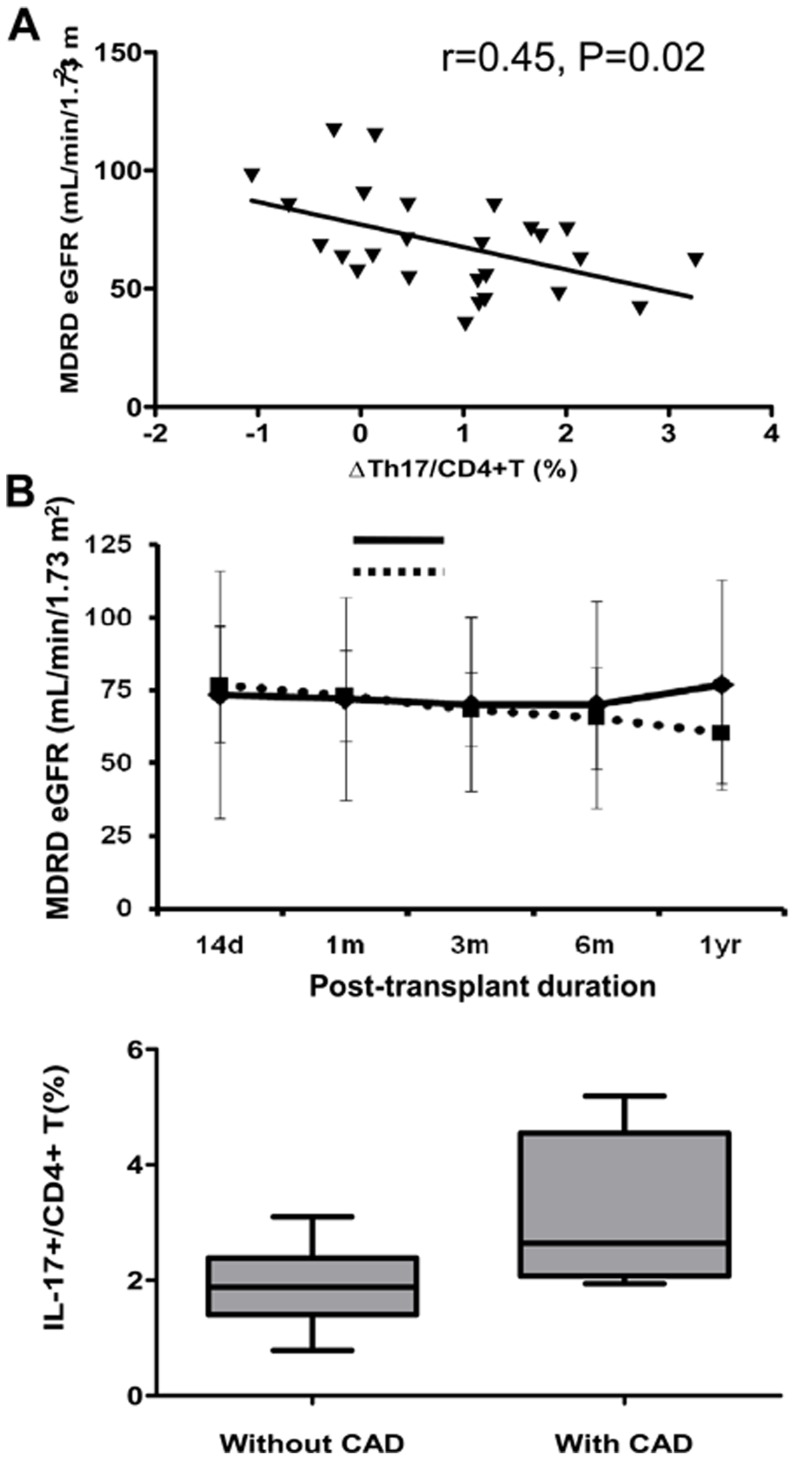
The clinical impact of the change of Th17 during early post-transplant period and long term follow up group. (A) ΔTh17/CD4+ T (%) was significantly associated with the allograft function at 1 year from KT (B) Patients who showed less increase of Th17 showed favorable allograft function compared to patients who showed more increase of Th17 during post-transplant 1 year. (C) Comparison between patients with CAD and without CAD. The percentage of Th17 was significantly decreased in patients without CAD. CAD, chronic allograft dysfunction; * *P*<0.05 vs. ΔTh17 high group. ^#^ *P*<0.05 vs. without CAD.

Figure 5 shows the association between Th17 and allograft function at 1 year after transplantation. It shows that the increase of Th17 after transplantation is negatively correlated with allograft function at 1 year post transplant (*r* = 0.45, *P* = 0.02) (Figure 5A). In contrast, Th1 (*r* = 0.00, *P* = 0.86) and Th2 (r = 0.00, P = 0.50) cells showed no significant correlation with allograft function 1 year post transplant. The median value of ΔTh17 was 1.08% (−1.06 to 3.26). The patients with ΔTh17>1.08 were designated the ΔTh17-high group and those with ΔTh17<1.08 were the ΔTh17-low group; comparison of the two groups showed that allograft function was significantly poorer in ΔTh17-high patients at 1 year post transplant (Figure 5B).

### Comparison of Th17 cell levels in patients with and without chronic allograft dysfunction (CAD)

In another cohort, which was at least 5 years after transplantation, we evaluated the proportion of Th1, Th2 and Th17 cells in the CD4^+^ T cells and compared them in patients with and without CAD. The serum creatinine (Scr) in the group without CAD (n = 8) was 0.9±0.1 mg/dL and the MDRD eGFR was 73.3±8.7 mL/min/1.73 m^2^. In the group with CAD (n = 8), Scr was 1.8±0.3 mg/dL (*P*<0.01 vs. patients without CAD) and MDRD eGFR was 37.0±14.0 mL/min/1.73 m^2^ (*P*<0.01 vs. patients without CAD). The duration of follow-up (9.6±1.9 years vs. 9.7±2.4 years, *P* = 0.88) and age at sampling (50.3±10.3 years vs. 48.9±11.4 years, *P* = 0.74) were matched between the two groups (Table 2). The percentage of Th17 cells was significantly higher in the group with CAD (3.1±1.3%) than in the group without CAD (1.9±0.7%) (*P*<0.01) (Figure 5C). In contrast, Th1 (16.5±7.0% vs. 19.5±10.5%, *P* = 0.38) and Th2 (1.8±1.0% vs. 1.7±1.4%, *P* = 0.85) showed no significant differences between the groups.

**Table 2:** Baseline characteristics of patients with long term follow-up.

	Without CAD (n = 8)	With CAD (n = 8)	P
**Age (year)**	50.3±10.3	48.9±11.4	0.74
**Male (n, %)**	3 (37.5)	5 (62.5)	0.31
**HLA mismatch number**	3.7±1.2	3.1±1.2	0.34
**Post-transplant duration** (year)	9.6±1.9	9.7±2.4	0.88
**MDRD eGFR**	73.3±8.7	37.0±14.0	<0.01
**Donor type (n, %)**			
Living related donor	2 (25.0)	4 (50.0)	0.60
Living unrelated donor	5 (62.5)	2 (25.0)	0.60
Deceased donor	1 (12.5)	2 (25.0)	0.60

CAD, chronic allograft dysfunction; MDRD eGFR. Modification of Diet in Renal Disease estimated glomerular filtration rate;

### Effects of Tac on Th1, Th2, Th17 and Treg subpopulations of CD4+ T lymphocytes from the peripheral blood of healthy donors and recipients early post transplant

**Figure 6:**
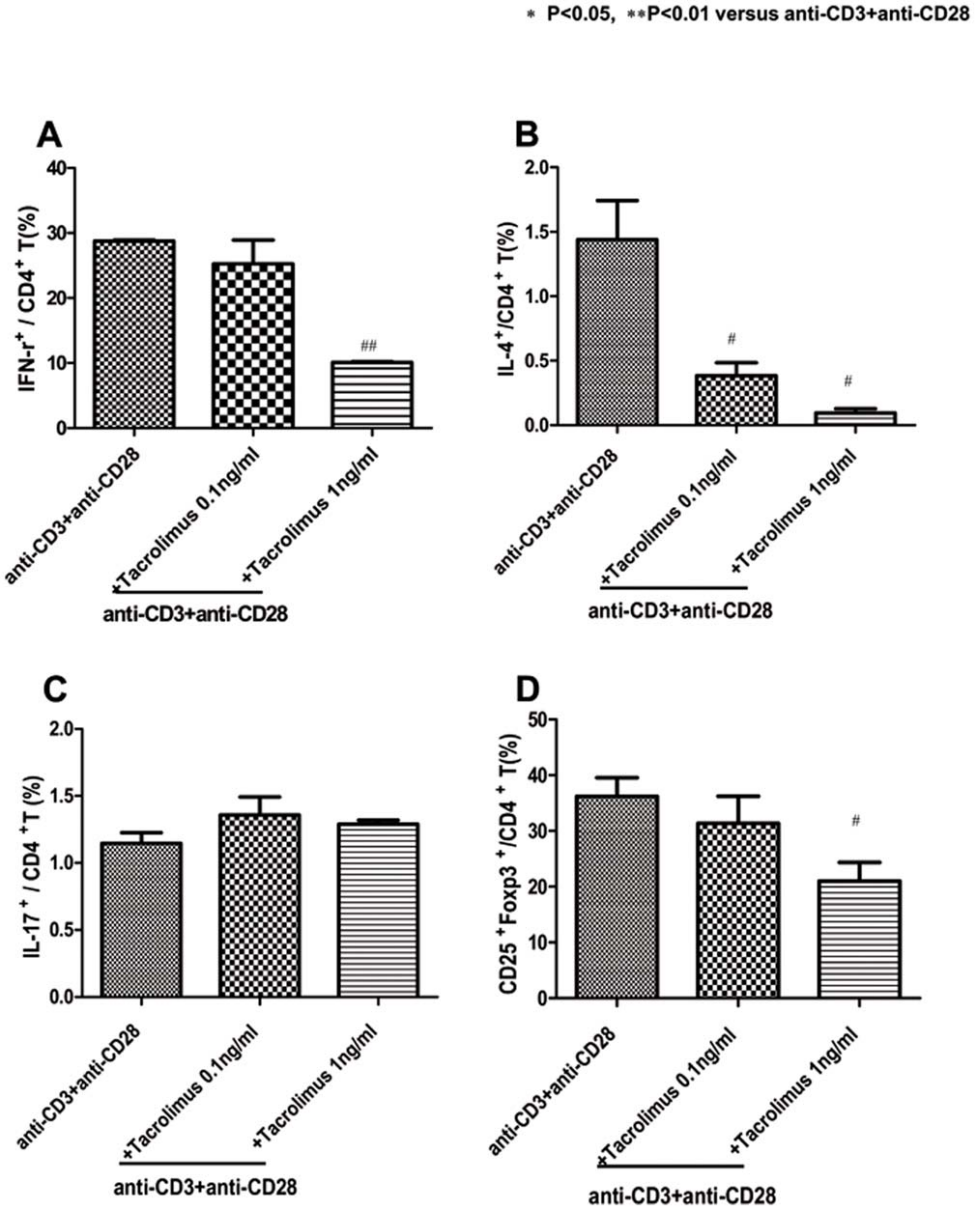
Effects of Tac in Th1, Th2, Th17 and Treg subpopulations of CD4+T lymphocytes from the peripheral blood of healthy donors. PBMC were preincubated for 1 h in the presence of Tac and stimulated with 1 µg/ml anti-CD3 and anti-CD28. Flow cytometry of intracellular IFN-r (A), IL-4 (B), Th17 (C) and Treg (D) in CD4+ T cells stimulated in the presence of plate-bound anti-CD3 plus anti-CD28, assessed after 48 h and then stimulated for 4 h with PMA and ionomycin in the presence of GolgiStop. The data are representative of three independent experiments. The values are expressed as the mean ± SEM. ^*^ *P*<0.05, ^**^ *P*<0.01 vs anti-CD3+anti-CD28. Tac, Tacrolimus.

**Figure 7:**
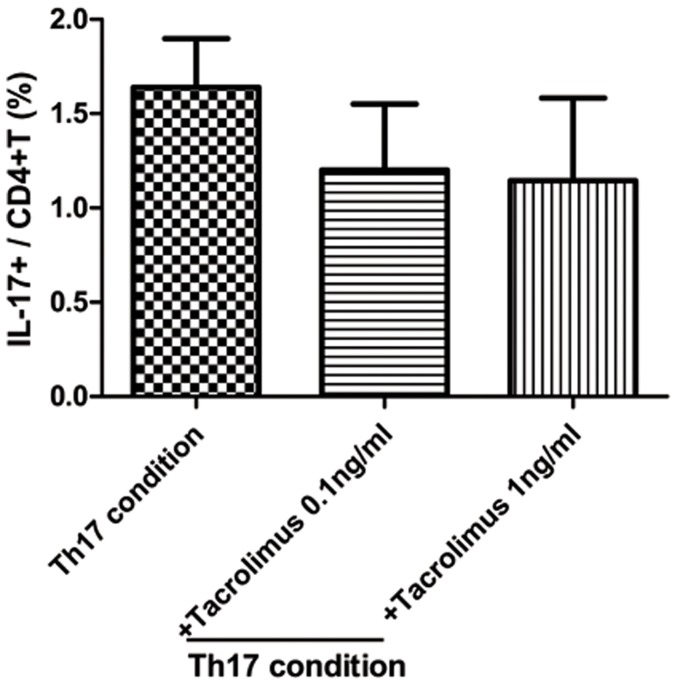
Effect of Tac in Th17 subpopulations of CD4+T lymphocytes from the peripheral blood of early-post transplant recipients. We used flow cytometry to examine how Tacrolimus regulates *in vitro* Th17 subpopulations of CD4+ T lymphocytes in Th17-polarizing condition. PBMC from renal transplant recipients were preincubated for 1 h in the presence of Tacrolimus and stimulated with Th17-polarizing condition for 48 h. Anti-CD3 (1 µg/ml), anti-CD28 (1 µg/ml), IL-1b (20 ng/ml), IL-6 (20 ng/ml) and IL-23 (20 ng/ml) were added to stimulate the differentiation of Th17 cells. Neutralizing antibodies to IFN-gamma (2 µg/ml) and IL-4 (2 µg/ml) were added in some experiments (R&D Systems). Flow cytometry of intracellular Th17 in CD4+ T cells stimulated in the presence of Th17-polarizing condition, assessed after 48 h and then stimulated for 4 h with PMA and ionomycin in the presence of GolgiStop. The data are representative of three independent experiments. The values are expressed as the mean ± SEM.

We used flow cytometry to examine how Tac regulates *in vitro* Th1, Th2, Th17 and Treg subpopulations of CD4+ T lymphocytes. PBMC were preincubated for 1 h in the presence of Tac and stimulated with 1 µg/ml anti-CD3 and anti-CD28 for 48 h. As shown in Figure 6A, the percentage of Th1 cells in the blood was significantly lower by Tac. The values were 28.7±0.3 (anti-CD3 and anti-CD28), 25.2±6.3% in the CD3, anti-CD28 and Tac 0.1 ng/ml (*P* = 0.429 compared with anti-CD3 and anti-CD28), 10.0±0.2% in the anti-CD3, anti-CD28 and Tac 1 ng/ml (*P* = 0.0001 compared with anti-CD3 and anti-CD28). As shown in Figure 6B, the percentage of Th2 cells in the blood was significantly lower in anti-CD3 and anti-CD28 cultured cells by Tac. The values were 1.4±0.5 (anti-CD3 and anti-CD28), 0.3±0.1% in the anti-CD3, anti-CD28 and Tac 0.1 ng/ml (*P* = 0.03 compared with anti-CD3 and anti-CD28), 0.09±0.05% in the anti-CD3, anti-CD28 and Tac 1 ng/ml (*P* = 0.03 compared with anti-CD3 and anti-CD28), and 0.01±0.004% in the anti-CD3, anti-CD28 and Tac 10 ng/ml (*P* = 0.009 compared with anti-CD3 and anti-CD28). By contrast, the frequency of Th17 cells did not differ significantly by Tac (anti-CD3, anti-CD28 and Tac 0.1 ng/ml, 1.3±0.2%, anti-CD3, anti-CD28 and Tac 1 ng/ml, 1.2±0.05%). The frequency of Treg cells was also significantly lower in the anti-CD3, anti-CD28 cultured cells by Tac. The values were 36.2±5.8 (anti-CD3 and anti-CD28), 31.3±8.4% in the anti-CD3, anti-CD28 and Tac 0.1 ng/ml (*P* = 0.09 compared with anti-CD3 and anti-CD28), 21.0±5.8% in the anti-CD3, anti-CD28 and Tac 1 ng/ml (*P* = 0.02 compared with the anti-CD3, anti-CD28). We tested the effect of Tac on Th17 in early-post transplant recipients in Th17 condition and Th17 was not suppressed in high doses of Tac (Figure 7).

## Discussion

This report is a prospective and sequential study investigating the immunologic parameters in a group of living-related renal allograft recipients who took Tac-based immune suppression. Most effector T cell subsets were decreased after transplantation because of the effect of the immune suppressant, but Th17 and IL-17-producing effector memory T cells were not suppressed and even showed a trend toward increasing after transplantation. This suggests that current immune suppressant treatments are not enough to suppress the alloimmune reaction, especially Th17-related immune responses.

Most immunosuppressive drugs have been selected and developed according to their ability to control T lymphocyte activation [3]. Under triple therapy composed of Tac, MMF and steroids, most T cell types can be efficiently suppressed. In addition, induction therapy using basiliximab, an anti-CD25 monoclonal antibody, can successfully prevent the robust proliferation of T cells [3]. Tac is effective in preventing Th1- and Th2-associated alloimmune responses, and addition of MMF can suppress humoral responses that are associated with the Th2 pathway [19], [20], [21]. In this study, total lymphocytes and CD4+ T cells did not show significant changes under immune suppressant treatment, but effector T cells, especially Th1 and Th2 cells, showed significant decreases at 1 month and 3 months after transplantation, consistent with previous reports.

About the effect of calcineurin inhibitor (CNI) on Th17 responses, only a small number of animal study has been conducted. In heart transplant animal model, CNI could not suppress Th-17 associated immune responses. [22]. The signature cytokines of Th1 and Th2 are both required to reciprocally reduce IL-17, while administration of antibody to either Th1 or Th2 cytokines increases IL-17 [23] As shown in previous reports, it is possible that administration of Th1-targeted Tac may not affect Th17 type T-cell responses, and rather increase Th17 reponses. However, little is known regarding the effect of immune suppressants on Th17-cell-mediated alloimmune responses in human.

Usually, the intensity of immune suppression is highest and the immune reaction to allograft most robust during the early posttransplant period. Therefore, we investigated the change in Th17 cells at 1 month and 3 months after transplantation compared with before transplantation. In contrast to other effector T cell subsets, the percentage of Th17 showed a gradual increase after transplantation, although the clinical course of the recipients was stable. In addition, even though the entire cohort of T_EM_ showed a significant decrease after transplantation, the proportion of IL-17-producing cells in T_EM_ cells significantly increased. This suggests that the increase in Th17 cells resulted from an increase in activated T cells, not of quiescent T cells.

We investigated the expression of markers associated with the development of Th17 cells, including IL-1beta and HMGB1. It is well known that IL-1beta plays an important role in the development of Th17 [24], while HMGB1 is a potent inducer of several proinflammatory cytokines, including IL-1beta and IL-6, which are considered crucial mediators in the induction of Th17 cells [25]. Our in vitro study showed that HMGB1 and IL-1beta expression was not suppressed after transplantation and even showed a gradual increase. This is consistent with the increased proportion of Th17 observed after transplantation by flowcytometry.

The clinical impact of Th17 cells in kidney transplantation has not been fully investigated. However, many previous reports suggested a role for Th17 in the progression of fibrosis in various organs. Fibrosis is a major component of the progression of chronic allograft injury in transplanted kidney [22], [26], [27], [28], [29], [30]. In kidney transplantation, Th17 cell infiltration hastens chronic rejection and fibrosis in allograft tissue by promoting lymphoid neogenesis [31]. In our previous report, we demonstrated that higher levels of infiltration of Th17 cells in renal allograft tissue are associated with more severe inflammation and chronic changes in the allograft tissue, and were significantly associated with the progression of interstitial fibrosis and tubular atrophy [32].

To investigate its clinical impact, we further evaluated the association between the changes in Th17 cells during the early posttransplant period and allograft function at 1 year after transplantation. The size of the change in Th17 was negatively correlated with MDRD eGFR at 1 year after transplantation, which suggests that chronic injury is more significant in patients who showed a greater increase in Th17 during the early posttransplant period. In addition, we investigated the significance of Th17 in another group of kidney transplant recipients with long-term follow-up. The CAD group showed a gradual deterioration in allograft function during follow-up, which suggests that chronic allograft injury may cause the allograft dysfunction. Interestingly, the proportion of Th17 cells was significantly increased in patients with CAD. It is possible that a sustained elevation in Th17 may be associated with the progression of chronic allograft injury through the mechanisms of both fibrosis and alloimmune reactions.

Finally, we tested the suppressive effect of Tac on Th1, Th2, Treg and Th17 cells. In kidney transplantation, only one study has shown that Tac can suppress Th17-related alloimmune responses in vitro [33]. However, the PBMC in that study were sampled from patients exposed to immune suppressants for a long time and who showed a stable clinical course. In contrast, we sampled peripheral blood from both healthy controls and kidney transplant recipients within 3 months after transplantation, at which time the most robust changes in immune response can develop. In healthy controls, Tac showed a dose-dependent suppressive effect on Th1/Th2. However, the suppressive effect of Tac on Th17 was not significant even with high doses of Tac. In the study using PBMC from recipients early post transplant, Th17 was not suppressed with most doses of Tac, which suggests that the suppressive effect of Tac on Th17 is inadequate. This is consistent with the results of the in vivo study, in which Th17 cells were not suppressed and even showed a tendency to increase, in spite of continued immune suppression including Tac.

In conclusion, Tac-based immune suppression could not suppress Th17 cell as well as other effector T cell subsets such as Th1 or Th2. The non-suppression of Th17 may be associated with the progression of chronic rejection even in stable renal allograft recipients. The results of this study might indicate a need to develop therapeutic options to suppress IL-17-producing effector T cells in renal transplant recipients.
